# Supplementary material for: MetaRibo-Seq measures translation in microbiomes
Source: Nat Commun. 2020 Jun 29;11:3268. doi: 10.1038/s41467-020-17081-z (PMC7324362; doi:10.1038/s41467-020-17081-z)
Supplement: Supplementary file 10 — Supplementary Data 7 [file 41467_2020_17081_MOESM10_ESM.zip › File2/Confidence_High_Taxonomy/94904_out.krona.html]

Javascript must be enabled to view this page.

members
magnitude
magnitudeUnassigned
count
unassigned
taxon
rank

94904\_out

2

2
2
superkingdom

phylum
1239
1

1
186801
class

1
186802
order

family
1
541000

genus
216851
1

853
1

SRS020271\_contig\_number\_contig-100\_7592.7593
species

phylum
1
1224

1
68525
subphylum

class
1
28221

order
213115
1

family
194924
1

genus
1
35832

species

SRS053356\_contig\_number\_6317
35833
1
